# Short and long-term reproductive outcomes after hysteroscopic adhesiolysis for infertile women

**DOI:** 10.5935/1518-0557.20220016

**Published:** 2023

**Authors:** Saeed Baradwan, Dalia Alharbi, Muhammad Salman Bashir, Ahmed Saleh, Dania Al- Jaroudi

**Affiliations:** 1 Department of Obstetrics and Gynecology, King Faisal Specialist Hospital and Research Centre, Jeddah, Saudi Arabia; 2 Department of Obstetrics and Gynecology, King Fahad Medical City, Riyadh, Saudi Arabia; 3 Research Services Administration, Department of Biostatistics, Research Center, King Fahad Medical City, Riyadh, Saudi Arabia; 4 Reproductive Endocrine and Infertility Medicine Department, King Fahad Medical City, Riyadh, Saudi Arabia

**Keywords:** Asherman syndrome, hysteroscopy adhesiolysis, infertility, pregnancy rate, live birth rate

## Abstract

**Objective:**

To evaluate reproductive outcomes after hysteroscopic adhesiolysis for patients with Asherman syndrome (AS) who presented with infertility and/or subfertility.

**Methods:**

A retrospective study was conducted in the Women’s Specialized Hospital, King Fahad Medical City, from December 2010 to December 2018. The medical records were reviewed for all infertile women who had hysteroscopic adhesiolysis. The specific study’s main reproductive outcomes included: [1] the overall rate of conception, [2] the overall rate of conception according to the severity degree of intrauterine adhesions (IUAs), [3] the reproductive methods for achieving conception, and [4] pregnancy outcomes. Reproductive methods for conception included spontaneous conception, ovulation induction (OI), intrauterine insemination (IUI), and in-vitro fertilization (IVF) with/without intracytoplasmic sperm injection (ICSI). Outcomes of pregnancy included ectopic pregnancy, miscarriage, and live birth events.

**Results:**

Forty-one patients (n=41) were analyzed. Their mean age was 32.2±4.6 years. The most common menstrual pattern amongst these patients was hypomenorrhea 46.4%. All patients resumed regular menstrual cycles after the adhesiolysis procedure. The overall conception rate during the 24 months follow up was 53.6%, and the overall live birth rate was 34.2%. Of the 22 patients who conceived, 12 patients (29.2%) conceived spontaneously, 2 (4.9%) with IUI, and 8 (19.5%) with IVF-ICSI. The patients with minimal IUAs had a significantly higher pregnancy rate (71.4%) when compared to those with moderate (47%) and severe (40%) IUA (two-tailed log-rank test, *p*=0.041).

**Conclusions:**

The spontaneous cumulative conception rate following hysteroscopic adhesiolysis was higher in patients with minimal IUAs than those with moderate and severe IUAs.

## INTRODUCTION

The Asherman syndrome (AS) was described by Joseph Asherman in 1948 as intrauterine adhesions (IUAs) obliterating partially or entirely the uterine cavity after trauma to the basal layer of the endometrium (Asherman, 1950). During the healing process, the traumatized opposing uterine walls adhere together by fibrotic process partially or completely (Di Guardo *et al*., 2020). Furthermore, depending on the degree of uterine cavity obliteration, patients with IUAs may be asymptomatic with normal menstrual cycles or symptomatic, presenting with menstrual abnormalities, such as hypomenorrhea or amenorrhea, infertility, recurrent pregnancy loss and abnormal placentation (Salazar *et al*., 2017; Dreisler & Kjer, 2019).

IUAs occur most frequently after dilatation and curettage for incomplete miscarriages (50%), postpartum hemorrhage (24%) and elective abortion (17.5%) (Diamond & Freeman, 2001; Salazar *et al*., 2017; Dreisler & Kjer, 2019). Furthermore, other etiological factors, include uterine surgery, i.e., Cesarean section, hysterotomy or myomectomy, and intrauterine infection, i.e., tuberculosis, can lead to intrauterine fibrosis and adhesion formation (Santamaria *et al*., 2018).

IUAs are classified as primary when occurring after pregnancy-related curettage or after hysteroscopic surgery. On the other hand, IUAs are classified as secondary when recurring at sites where adhesiolysis had been performed (AAGL Elevating Gynecologic Surgery, 2017). IUAs can lead to uterine factor subfertility or infertility, and this may be due to the formation of endometrial fibrosis affecting implantation or uterine cavity obliteration.

Direct visualization of the uterus via hysteroscopy is the gold standard and most reliable method for diagnosis and treatment of IUAs (Khan & Goldberg, 2018). Hysteroscopic adhesiolysis is well known for being the procedure of choice for the treatment of IUAs (Salazar *et al*., 2017; Santamaria *et al*., 2018; Dreisler & Kjer, 2019). Treatment aims to restore normal endometrial cavity shape and functions. Several studies reported overall restoration of normal menstrual cycles in 70-82%, the conception rates were 45-97%, and the term delivery rate in women who achieved pregnancy was 25-80% (Yamamoto *et al*., 2013; Song *et al*., 2014; Bhandari *et al*., 2015; di Spiezio Sardo *et al*., 2016; Di Guardo *et al*., 2020).

The aim of this study was to evaluate reproductive outcomes after hysteroscopic adhesiolysis for women who presented with infertility and/or subfertility.

## MATERIALS AND METHODS

### Patients

In this retrospective study, medical records were reviewed from December 2010 to December 2018, for all infertile women who attended the Reproductive Endocrine and Infertility Medicine Department at the Women’s Specialized Hospital, King Fahad Medical City, Riyadh, Saudi Arabia. We included women aged 20-40 years who presented with infertility and/or recurrent pregnancy loss (≥2 spontaneous miscarriages) with suspected or confirmed diagnosis of IUAs. We employed a purposive sampling technique in this study.

All patients underwent preoperative evaluations, including a detailed history of menstrual pattern for the last six cycles, previous intrauterine surgery, reproductive history, types and duration of infertility, tubal patency test, and spouse’s semen analysis. The patient’s age, height, weight, body mass index (BMI) were recorded and analyzed. Blood investigations including serum hormone measurements that regulate menstrual cycle rhythm, luteinizing hormone (LH), follicle-stimulating hormone (FSH), estradiol (E2), androgens, thyroid stimulating hormone (TSH) and prolactin were made on the follicular phase of the menstrual cycle or at a randomly chosen time in patients with amenorrhea.

Patients over 40 years or with male factor infertility, premature ovarian failure (FSH >40 U/L), cycle irregularity due to polycystic ovarian syndrome, an abnormal hormonal profile that might affect reproductive success, or any other uterine malformation, such as fibroid uterus, uterine anomalies or bilaterally blocked tubes, and prior history of hysteroscopic adhesiolysis were excluded from the study. Hypomenorrhea was defined as short or scanty periods, and amenorrhea as absent menstrual cycles for ≥6 months.

### Classification

All cases were diagnosed by hysteroscopy and classified according to March’s classification of IUAs (mild if filmy adhesion occupying less than one-quarter of the uterine cavity and ostial areas and the upper fundus is minimally involved or clear; moderate if one-fourth to three-fourth of the cavity is involved and ostial areas and upper fundus partially involved and no agglutination of uterine walls; or severe if more than three-fourth of the cavity is involved; and occlusion of both ostial areas and upper fundus and agglutination of uterine walls) (March & Israel, 1976; Manchanda *et al*., 2021).

### Procedure

Hysteroscopic adhesiolysis was performed for all patients by four experienced endoscopic surgeons in the department using similar techniques and under general anesthesia. The cervix was initially dilated to “9” using Hegar’s dilators. An 8-mm, 12° rigid telescope (Karl Storz, Tuttlingen, Germany), using bipolar electrode needle or loop, cutting current setting of 60 W and 50 W for coagulation (Sabre 2400; CONMED, New York, NY, USA). Normal saline was used as a distending medium for all procedures. Adhesiolysis dissections began inferiorly then anteriorly towards the fundus until the pink myometrium was visible. Both tubal ostia were visualized, and a panoramic view of the cavity was obtained. Concomitant laparoscopy was performed in 15 patients, and transabdominal B-mode ultrasound sonography, also used in 4 patients to guide the surgeon during the difficult procedures. This was to prevent uterine perforation and to confirm tubal patency in patients in whom the tubal factor was suspected. Cefazolin sodium antibiotic (2 g) was administered to all patients as prophylaxis at the start of the procedure.

Postoperatively, all patients were given cyclical hormonal therapy for three months, estradiol valerate 6 mg per day in divided and Norethisterone acetate (Primolut N) 5 mg three times a day for the last seven days of the cycle. The patients were followed up in the clinic every 3 months for a period of 24 months post procedure.

### Reproductive outcomes

The specific study’s main reproductive outcomes included: [1] the overall rate of conception/pregnancy, [2] the overall rate of conception according to the severity degree of IUAs, [3] the reproductive methods of achieving conception/pregnancy, and [4] the outcomes of the pregnancy. Reproductive methods of conception included spontaneous conception, ovulation induction (OI), intrauterine insemination (IUI), and in vitro fertilization (IVF) with or without intracytoplasmic sperm injection (ICSI). Outcomes of pregnancy included ectopic pregnancy, miscarriage, and live birth events. Live birth was defined as delivery of a live fetus weighing >500 g that resulted in at least one live neonate born.

### Statistical analysis

All data were entered and analyzed through the Statistical Package for Social Sciences (SPSS) software, version 25, for Windows (IBM Inc., Armonk, NY, USA). The results were presented as mean ± standard deviation (SD) and ranges for continuous variables and frequency (percentages) for categorical variables. Since the sample size was small, all percentages were supplemented with 95% confidence intervals (CIs), calculated in the SPSS according to the Clopper-Pearson (exact) method. The cumulative conception rate was evaluated using the Kaplan-Meier survival analysis and the statistical significance was evaluated according to the log-rank test. A two-tailed *p*-value <0.05 was considered statistically significant.

### Ethical approval

The Institutional review board granted ethical approval for this study at King Fahad Medical City (Institutional Review Board [IRB] log no. 19-024). Routine informed consent was obtained for any research use during hospitalization.

## RESULTS

The data for 41 women were available for analysis and they were followed up for 3-24 months. Their ages ranged between 21 and 39 years, with a mean of 32.2 ±4.6 years (95% CI: 30.79-33.70). Their BMIs ranged between 23.4 and 38.1 kg/m^2^, with a mean of 29.07 ± 3.60 kg/m^2^ (95% CI: 27.9-30.2). The patients’ previous history and preoperative menstrual pattern presentations are shown in Table 1. The most common menstrual pattern amongst these patients was hypomenorrhea (n=19/41, 46.4%, 95% CI: 30.7- 62.6) (Table 1).

**Table 1:** Clinical presentation and previous history of the patients (n=41).

Characteristics	n (%), [95% CI]
Menstrual pattern Normal Hypomenorrhea Amenorrhea	16 (39.0), [24.2-55.5] 19 (46.4), [30.7-62.6] 6 (14.6), [5.6-29.2]
Dysmenorrhea	26 (63.4), [46.9-77.9]
Infertility type Primary Secondary	12 (29.3), [16.1-45.5] 29 (70.7), [54.5-83.9]
Previous history of miscarriage	23 (56.1), [39.7-71.5]
Previous history of curettage	21 (51.2), [35.1-67.1]
Previous history of postpartum hemorrhage	13 (31.7), [18.1-48.1]
Previous history of caesarean delivery	15 (36.6), [22.1-53.1]

CI: confidence interval; n: sample size

Details of hysteroscopic procedures performed and intraoperative findings are shown in Table 2. There were no complications reported with any of the surgical procedures. All patients resumed normal menstrual cycles after the adhesiolysis. The overall conception rate during 24 months follow up was 53.6% (n=22/41, 95% CI: 37.4-69.3) and the overall live birth rate was 34.2% (n=14/41, 95% CI: 20.1-50.6). Of 22 patients who conceived, 12 patients conceived spontaneously (80%, 95% CI: 51.9-95.7), two patients conceived with IUI from the husband (50%, 95% CI: 6.8-93.2), eight patients conceived with IVF-ICSI (44.4%, 95% CI: 21.5-69.2) and none (0%) following ovulation induction and time intercourse (Table 3). Among the 22 pregnant women, eight patients had pregnancy loss (36.4%, 95% CI: 17.2-59.3), six women had a first-trimester miscarriage (27.3%, 95% CI: 10.7-50.2), and two women had an ectopic pregnancy (9.1%, 95% CI: 1.1-29.2) (Table 3).

**Table 2:** Operative procedures and intraoperative findings of the patients (n=41).

Variables	n (%), [95% CI]
Operative procedure Hysteroscopy alone Hysteroscopy and ultrasound Hysteroscopy and laparoscopy	22 (53.7), [37.4-69.3] 4 (9.7), [27-23.1] 15 (36.6), [22.1-53.1]
Stage of March classification Minimal Moderate Severe	14 (34.1), [20.1-50.6] 17 (41.5), [26.3-57.9] 10 (24.4), [12.4-40.3]

CI: confidence interval; n: sample size

**Table 3:** Operative procedures and intraoperative findings of the patients (n=41).

Variables	N	Pregnancy n (%), [95% CI]	Ectopic n (%), [95% CI]	Miscarriages n (%), [95% CI]	Live birth n (%), [95% CI]
Spontaneous	15	12 (80), [51.9-95.7]	1 (6.7), [0.2-31.9]	3 (20), [4.3-48.1]	8 (53.3), [26.6-78.7]
OI	4	0	0	0	0
IUI	4	2 (50), [6.8-93.2]	1 (25), [0.6-80.6]	0	1 (25), [0.6-80.6]
IVF-ICSI	18	8 (44.4), [21.5-69.2]	0	3 (16.7), [3.6-41.4]	5 (27.8), [9.7-53.5]

CI: confidence interval; IUI: intrauterine insemination; IVF-ICSI: in vitro fertilization-intracytoplasmic sperm injection; OI: ovulation induction; N: number of patients tried; n: sample size

**Figure 1:**
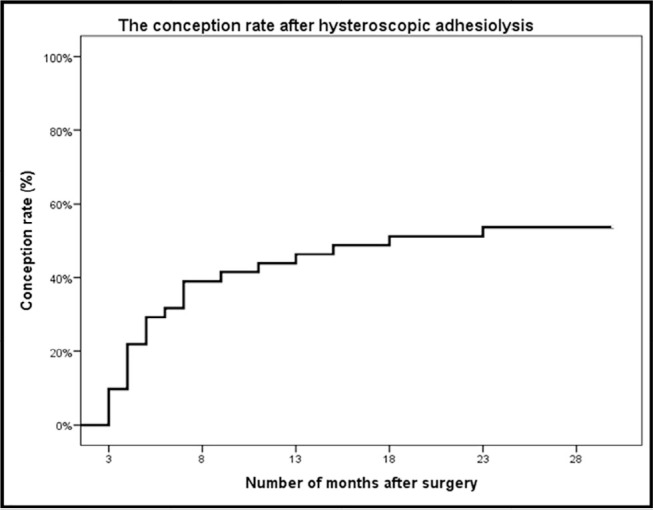
The conception rate after hysteroscopic adhesiolysis (n=41).

**Figure 2:**
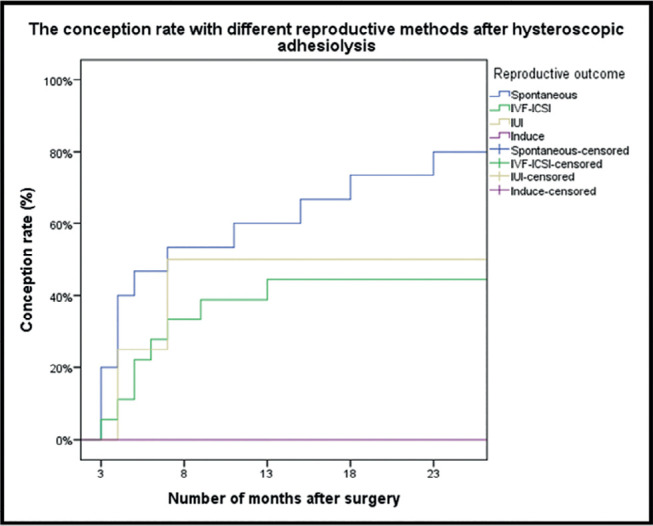
The conception rate with different reproductive methods after hysteroscopic adhesiolysis (n=41).

**Figure 3:**
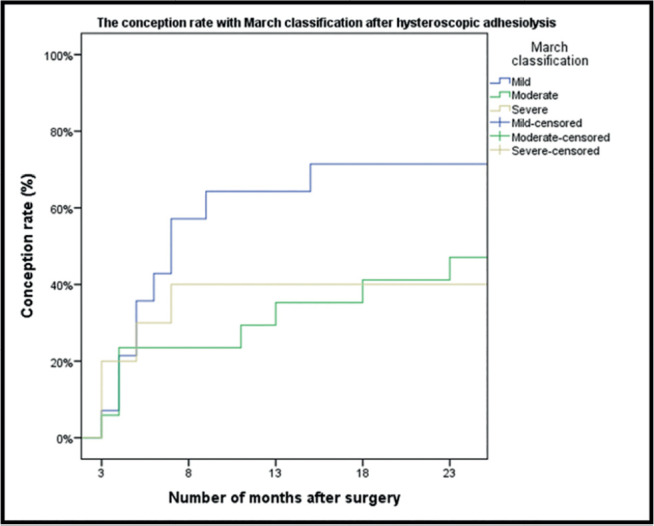
The conception rate with March classification after hysteroscopic adhesiolysis (n=41).

There was no significant association between conception rate and preoperative menstrual pattern. The total cumulative conception rate for patients who conceived was calculated as a 1-year survival rate and it is shown in Figure 1. Further analysis with regards to the method of achieving pregnancy, spontaneous pregnancy occurred in 80% of patients (95% CI: 51.9-95.7) which was significantly higher than that for patients with IUI (50%, 95% CI: 6.8-93.2) and IVF-ICSI (44.4%, 95% CI: 21.5-69.2), (two-tailed log-rank test, *p*=0.038) (Figure 2). Analysis of the cumulative conception rate revealed that patients with minimal IUAs had a significantly higher pregnancy rate (n=10/14, 71.4%, 95% CI: 41.9-91.6) compared with those who had moderate (n=8/17, 47.1%, 95% CI: 23.0-72.2) and severe (n=4/10, 40%, 95% CI:12.2-73.8) IUAs (two-tailed log-rank test, *p*<0.041) (Figure 3).

## DISCUSSION

Diagnosis of AS has increased due to the availability of modern advanced imaging and hysteroscopy experts able to make accurate assessments of the condition. The majority of patients with AS present with infertility (Salazar *et al*., 2017; Dreisler & Kjer, 2019; Di Guardo *et al*., 2020), and thus hysteroscopic adhesiolysis of IUAs is usually deemed necessary in women with infertility and recurrent pregnancy loss.

In the present study, we analyzed 41 women who presented with infertility and/or subfertility due to variable degrees of IUAs. The success rates for resuming normal menstrual cycles following hysteroscopic adhesiolysis was reported to be 70-90% (Thomson *et al*., 2007; Roge *et al*., 1997; Yu *et al*., 2008; Yamamoto *et al*., 2013; Song *et al*., 2014; Bhandari *et al*., 2015; di Spiezio Sardo *et al*., 2016; AAGL Elevating Gynecologic Surgery, 2017; Chen *et al*., 2017; Di Guardo *et al*., 2020). In the current study, all patient’s normal menstrual flow returned following the adhesiolysis, since the main purpose of treating infertile women with AS is to improve both conception and live birth rates. Several studies have evaluated reproductive outcomes after hysteroscopic adhesiolysis. Pregnancy rates have been reported between 25-76% and live birth rates 64-79% (Yamamoto *et al*., 2013; Yu *et al*., 2008; Bhandari *et al*., 2015). Our study showed that the overall pregnancy rates were 53.6% (95% CI: 37.4-69.3), and live birth rates were 34.2% (95% CI: 20.1-50.6) during 24 months of follow up. The conception rate in the present study was similar to that reported by Roge *et al*. (1997) (54%), and the live birth rate in our study was similar to that reported by Yamamoto *et al.* (2013), which was around 33.3%.

Several prognostic factors affecting conception and live birth rates following hysteroscopic adhesiolysis have been investigated, including adhesion severity (Di Guardo *et al*., 2020). In our study, we further analyzed pregnancy rate results according to the severity of IUAs categorized into minimal, moderate, and severe cases. The pregnancy rates were 71.4% (95% CI: 41.9-72.2), 47% (95% CI: 23.0-72.2), and 40% (95% CI: 12.2-73.8), respectively. This agrees with previous reports that pregnancy rates decreased as the severity of the adhesions increased (30-40%) in severe IUAs cases (Thomson *et al*., 2007; Roge *et al*., 1997; Pabuçcu *et al*., 1997). This result suggests that adhesion severity in our study was the main factor that had a negative correlation with conception. This consequential lower live birth rate results associated with severe IUAs cases can be explained by the presence of endometrial atrophy, which has a detrimental effect on implantation and adhesion formation (Roge *et al*., 1997; Dreisler & Kjer, 2019).

A limitation of our study was the small sample size; however, our results agreed with the literature. Moreover, a limitation of our study was the lack of a second-look hysteroscopy in all patients. Therefore, patients who did not conceive (46%), could have re-adhesions that would need a second procedure. It is well known that severe cases of IUAs have a high rate of re-adhesions after adhesiolysis 20-62.5% (Pabuçcu *et al*., 1997; Capella-Allouc *et al*., 1999; Preutthipan & Linasmita, 2000; Di Guardo *et al*., 2020). This suggests that second look and second adhesiolysis procedures may be required in selected non-pregnant or amenorrheic cases, to increase pregnancy and live birth rates. This observation from our study is contradictory to Wenzhi *et al*., who recommend routine early second-look hysteroscopy to all patients within two months from the first procedure (Xu *et al*., 2018). To reduce the likelihood of adhesion reformation, all patients in our series received postoperative antibiotics and estrogen-progesterone hormonal therapy. This agrees with Salma *et al*. (2014), who stated that the postoperative use of an intrauterine device (IUD) has no advantage over antibiotics and hormonal treatment. Additionally, a recent meta-analysis concluded that there was no evidence for higher pregnancy and live birth rates by using any barrier gel following operative hysteroscopy (Bosteels *et al*., 2014).

The cumulative live birth for women with a normal cavity after three cycles of IVF has been reported between 45-53% (Malizia *et al*., 2013). In our study, we found the live birth following IVF for 18 women with AS to be 27.8%, and the conception rate to be 44.4%. Due to the small sample size of our patients, we could not adequately assess the impact of age on the reproductive outcomes. Nevertheless, the existing body of literature highlights that pregnancy rates in IVF are significantly related to age. More specifically, advances in age from both males as well as females have been depicted to correlate with poor pregnancy outcomes (Tan *et al*., 2014; Ubaldi *et al*., 2019; Van Opstal *et al*., 2021).

To the best of our knowledge, there are no guidelines or recommendations to prefer one method of assisted reproductive techniques over another in AS. In light of the study results, we recommend conducting a study with a larger and adequate sample size to increase the study power and thus provide more generalizable results.

## CONCLUSION

The spontaneous cumulative conception and live birth rates following hysteroscopic adhesiolysis were higher in patients with minimal IUAs than those with moderate and severe IUAs. Patients with severe adhesions, who do not resume normal menstrual cycles and/or do not conceive, may be offered a second-look hysteroscopy with a possible second adhesiolysis procedure.
